# Massachusetts Dental Society

**Published:** 1897-10

**Authors:** 

**Affiliations:** Massachusetts Dental Society


### MASSACHUSETTS DENTAL SOCIETY.

A MEETING of the Massachusetts Dental Society was held on Wednesday, June 2, 1897, at the Harvard Dental College building, Boston, the President, Dr. Waldo E. Boardman, in the chair. After the routine business, Mr. James L. Gethins read a paper on "Electricity and Dentistry."

(For Mr. Gethin's paper, see page 631.)

### DISCUSSION.

*The President.*—The subject is now open for discussion.

*Dr. Willard D. Ball.*—Mr. President, I would like to know what the difference would be between the storage battery and wet cell battery.

*Mr. Gethins.*—The storage battery is, as you may say, a reservoir of electricity. There is practically no resistance, and you can use a less number of cells in doing the work. That is the reason the storage battery is superior to the other.

*Dr. S. W. Thayer.*—What is estimated to be the ability of the storage battery to deliver,—that is, how much can be delivered with safety and leave some in the storage battery?

*Mr. Gethins.*—I should say that never more than three-fourths of discharge of the actual capacity in ampères should be taken out of the battery. If you have eighty ampères, not more than sixty should be taken out.

*Dr. Thayer.*—That would leave a normal quantity, enough to preserve the plates, not drawn out?

*Mr. Gethins.*—Yes.

*The President.*—The chair desires to state that he has two storage batteries of the Sorley make that have been used for the last five years, now in the sixth year. They are apparently in as good condition as ever. Adding a little sulphuric acid and water, once in a year and a half, provides for evaporation.

*Mr. Gethins.*—I should like to ask a question of any gentleman here who may have actually tested the question of current used in cataphoresis, if he has ever actually used a very fine recording metre? My test has been so far with voltage, and I want to see whether six is required or sixty, and I think that I have practically determined that. Speaking of that, I would like to say one thing more. It seems to me, from an electrical stand-point, that in applying cataphoresis a great deal depends upon the contact. Take, for instance, the employment of the hand for contact with the cathode, the current has got so much farther to go.

*The President.*—Dr. Piper has studied this subject some months, and perhaps he would like to say something upon it.

*Dr. James R. Piper.*—I have been using an apparatus which I constructed myself out of Mesco's dry batteries. I have in a box twelve of these, and they are turned on by means of a switch, one at a time, but the points of contact are so arranged that the switch is on two at the same time, and if you take it off of one it does not break the current. I have used it now for eight months with a great deal of satisfaction, and cannot see but that it does its work just as satisfactorily as some more expensive apparatus.

*Mr. Gethins.*—I would like to ask Dr. Piper how many cells he has found most effective.

*Dr. Piper.*—I use from three to six.

*The President.*—Does any other person desire to ask a question?

*Dr. Fessler.*—I do not know that it comes directly under the discussion, but I would ask, if cocaine is carried into the cavity, what difference how high the current or what the voltage may be?

*Mr. Gethins.*—From an electrical stand-point, I should say that the voltage is simply like a trip-hammer driving down on that tooth; and, as to the matter of saturation, if you cannot thoroughly insulate that tooth, it may saturate the whole jaw. I have no doubt we can saturate, to come back to the storage battery, any quantity of lead plate simply by using a quantity of current; but it does not make a particle of difference, in that particular case, whether we have ten thousand volts or two and a half volts. The only result I can see electrically would be a very *disagreeable sensation* to the patient by using *high voltage.*

*Dr. Thayer.*—Mr. Chairman, Mr. Gethins inquired, I think, how much current the gentlemen have been in the habit of using in cataphoresis to get anæsthesia,—is that the question?

*Mr. Gethins.*—Yes.

*Dr. Thayer.*—I have seen quite a number of instruments, and I think those most effective which one can use intelligently are those which have attached to them a galvanometer. You can measure and know what amount or quantity you can get; and the largest quantity that I have seen or found to be required to extract teeth which have calcareous matter interspersed—it is very difficult to produce anæsthesia in these cases—is five-tenths of a milliampère, and I have seen anæsthesia produced inside of five minutes.

*Mr. Gethins.*—I would like to ask the doctor if I am right in my supposition that the time required to accomplish this result depends a great deal upon the actual service of contact at the tooth?

*Dr. Thayer.*—Of course, the contact has a great deal to do with it.

*The President.*—Has Dr. Strout done any experimenting in the way of cataphoresis?

*Dr. B. H. Strout.*—My experiments have been limited, and I do not care to make any report now. I am keeping a record of what I am doing, and may make one later.

*The President.*—If there are no further remarks on the subject, I will ask Mr. Gethins to close the discussion.

*Mr. Gethins.*—I do not know that I have much more to say. There is one question which a gentleman asked me about the effect of higher voltage. The resistance of the gum that surrounds the teeth governs to some extent the actual amount of current that goes through, and I can readily appreciate the fact that there may be portions of that gum of higher resistance than others, and a higher voltage may tend to drive a larger amount of current around that place. Resistance is overcome by voltage.

In reference to a question that may be asked, how storage batteries may be charged, I have found the most satisfactory method to be by means of an improved form of gravity battery of my invention, which is simply left constantly connected to the storage cells, charging them at a low rate. This keeps the storage cells in first-class condition, and always ready for immediate use.

The life of a charging battery is six months or more without attention, and expense of maintenance about two cents a day.

Henry L. Upham, D.M.D.,
*Editor Massachusetts Dental Society.*

